# Predation impacts of invasive raccoons on rare native species

**DOI:** 10.1038/s41598-020-77016-y

**Published:** 2020-11-30

**Authors:** Sakura Oe, Mariko Sashika, Ayako Fujimoto, Michito Shimozuru, Toshio Tsubota

**Affiliations:** 1 Laboratory of Wildlife Biology and Medicine, Faculty of Veterinary Medicine, Hokkaido University, Kita 18 Nishi 9, Kita-ku, Sapporo, Hokkaido 060-0818 Japan; 2 Raccoon Researchers Group, Kita 21 Nishi 3, Kita-ku, Sapporo, Hokkaido 001-0021 Japan

**Keywords:** Conservation biology, Invasive species, Stable isotope analysis

## Abstract

In Japan, there are concerns that invasive alien raccoons prey on rare native species during their spawning season from late winter to early summer. We investigated raccoon predation impact by examining the predation presence using DNA metabarcoding and extent of predation on rare native species using stable isotope analysis. We captured raccoons in Hokkaido, Japan from April to August in 2018 and 2019. We analysed raccoon faeces and gastric contents by DNA metabarcoding to detect the rare native Hokkaido salamander and Japanese crayfish. Hokkaido salamanders were detected from gastric contents, but Japanese crayfish were not detected in any samples. Stable isotope analysis of raccoon muscle samples and the Bayesian mixing model were used to estimate each food resource’s contribution to the raccoon diet. Animal food resources accounted for 70% of total consumed food. The foraging ratios of amphibians and crustaceans were about 9% and 5%, respectively. Raccoons have been found to use amphibians at a higher rate than previously reported, including a rare endangered species, the Hokkaido salamander. Hokkaido salamander and Japanese crayfish spawn in the spring, and increased predation pressure by raccoons may directly impact populations of these rare native species.

## Introduction

Invasive alien species are species whose introduction and/or spread outside their natural habitats threaten biological diversity^1,2^. They are considered one of the most significant causes of extinction and decline of wild native species. Since the seventeenth century, invasive alien species have contributed to nearly 40% of all animal extinctions for which the cause is known^2^. In particular, invasive alien mammals are thought to have serious impacts on native ecosystems because of their high trophic level. Therefore, it is important to analyse the feeding habits of alien species if they are preying on rare native species^1^.

Raccoons (*Procyon lotor*) represent one of the most problematic invasive alien species in Japan^1^. Raccoons are medium-sized mammals belonging to the Procyonidae family in the Carnivora, and they are native to North America^3^. Raccoons are omnivorous and known to diversify their diet according to the food resources available in each region^4^. However, they are known to prefer aquatic organisms such as amphibians, small fish, and crayfish^5^. In North America, animal food resources are generally more important from late winter to spring when plant food resources are deficient^6–8^. In Japan, raccoons became popular pets because of the influence of television cartoons in the 1970s, and many were imported from North America^9,10^. However, many pet raccoons were abandoned or released into the wild by their owners^9,10^, and some raccoons escaped from captivity on their own. Consequently, wild raccoons are now distributed throughout Japan^9,10^. Wild raccoons negatively affect agriculture, human health (via transmission of infectious diseases), and the ecosystem. Impacts on the ecosystem include competition with native species and direct predation on rare native species^9^.

It is difficult to evaluate the extent of predation on rare native species by raccoons. This is because it is very unlikely that rare native species whose populations have already declined will be present in stomach contents and faeces^11^. Rare native species that are threatened by raccoon predation in Hokkaido include Hokkaido salamander (*Hynobius retardatus*) and Japanese crayfish (*Cambaroides japonicus*)^9^. Hokkaido salamander is an endemic Japanese species that inhabits only Hokkaido. The populations in Ishikari and Tokachi subprefectures are listed as endangered “Local Populations (Lp)” in the Hokkaido Red List^12^. However, information on its conservation status is insufficient in the Japanese Ministry of the Environment's Red List. Therefore, an ecological survey of Hokkaido salamander is required. Japanese crayfish is an endemic species of Japan that inhabits only Hokkaido, Aomori, Akita, and Iwate prefectures, and is listed as a vulnerable species (VU) in the Red List of the Ministry of the Environment of Japan^13^.

**Figure 1:**
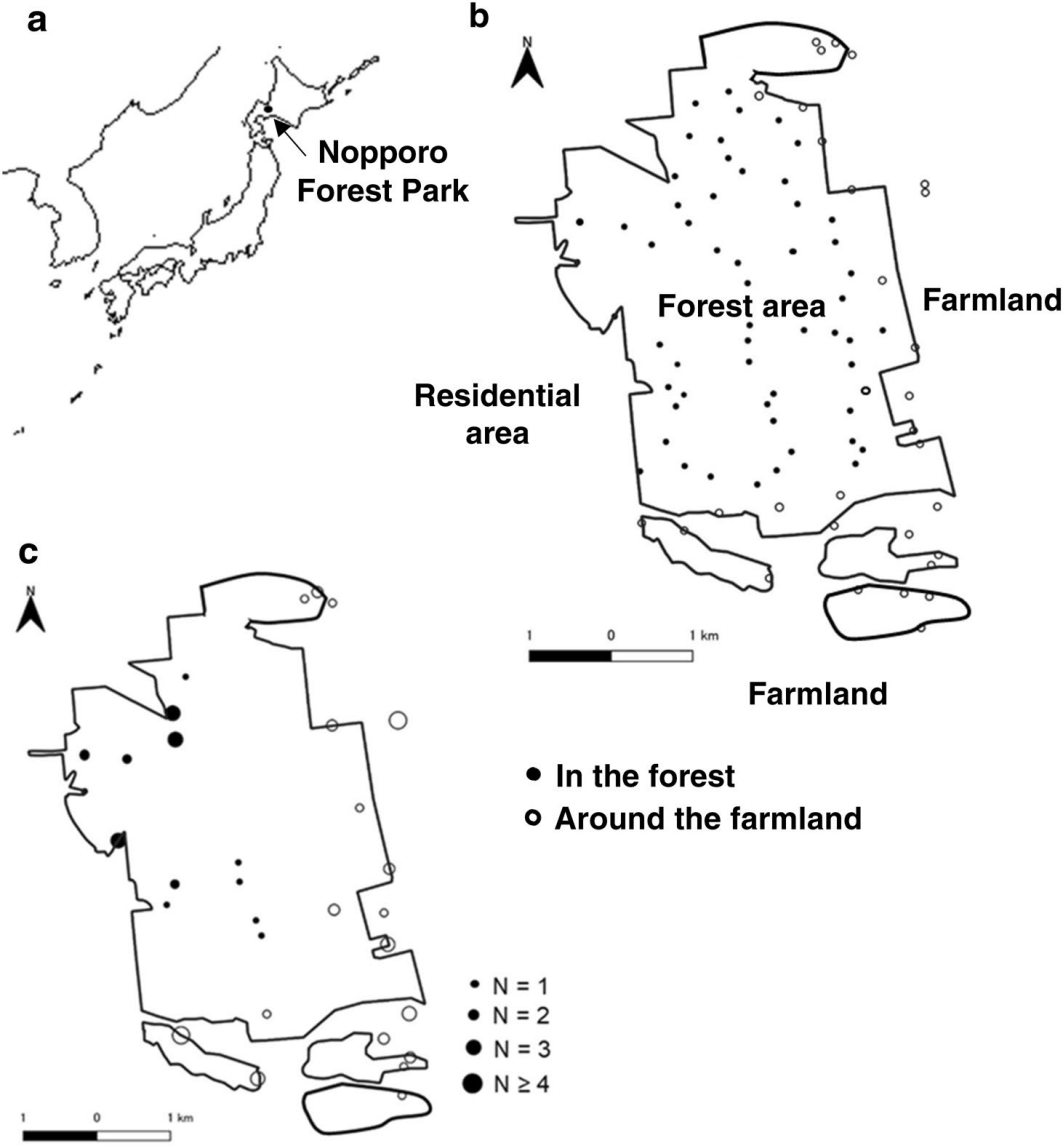
Map of Nopporo Forest Park and capture sites of raccoons. (**a**) Map of Nopporo Forest Park, central Hokkaido, Japan. (**b**) Map showing trapping points in Nopporo Forest Park. Open circles show trapping points around the farmland (31 points) and closed circles show those in the forest (55 points). (**c**) Capture sites of raccoons in 2018 and 2019. Circle size is proportional to number of captured raccoons. Figure was developed using data from National Land Numerical Information (Administrative Zones, Natural Park) and edited by us. Publication of the figure under a CC BY license was permitted by the National Spatial Planning and Regional Policy Bureau, MLIT of Japan, copyright 1974–2018.

To develop appropriate conservation strategies, it is important to understand the population decline of Hokkaido salamander and Japanese crayfish, including the impacts of predation by raccoons. To date, crayfish has been confirmed as prey for raccoons, based on analyses of the gastrointestinal contents of multiple raccoons^14^. However, little is known about the extent of predation by raccoons on Japanese crayfish. Since the invasion of raccoons at our study site, Nopporo Forest Park (Fig. 1), the remains of Hokkaido salamanders that may have been preyed on by raccoons have been repeatedly found from April to early May, and a local reduction in the number of Hokkaido salamander egg sacs has been confirmed^15^. Regarding the possibility that other native medium-sized mammals inhabiting the forest may prey on Hokkaido salamander, camera trap surveys have not detected foraging of salamanders by Hokkaido raccoon dogs (*Nyctereutes procyonoides albus*) or Hokkaido red foxes (*Vulpes vulpes schrencki*)^15^. The spawning season of Hokkaido salamander is in spring, from April to May, when many males gather to fertilise the egg sacs^16^. Because raccoon food resources are depleted from late winter to early summer, Hokkaido salamander may be a food resource for raccoons. However, the remains of Hokkaido salamander have never been detected in raccoon stomach contents or faeces because they are easily and fully digested.

Stable isotope analysis is useful in such cases. In recent years, measurement of stable isotopes has become a widespread tool for ecologists to estimate community structure and ecosystem function. The δ^13^C and δ^15^N values are commonly used in wildlife feeding habit analyses; δ^15^N increases from 3 to 4‰ with one trophic level increase. Therefore, the δ^15^N value reflects the trophic position of organisms in the food web^17^. Conversely, δ^13^C does not change much with trophic position (0–1.5‰)^18–20^. The δ^34^S reflects underlying local bedrock, atmospheric deposition, and microbial processes active in soils. Therefore, δ^34^S in animal tissues can be used to distinguish local food webs from extra-local (migrants) individuals in a region because extra-local individuals have different environmental δ^34^S values. In addition, δ^34^S values provide supplementary information to δ^13^C and δ^15^N data^21^. The diet proportions for consumers in a food web based on stable isotope information can be estimated using the Bayesian mixing model through IsoWeb^22^.

There are concerns that invasive alien raccoons prey on rare native species during their spawning season in Japan^15^; however, there was little available information about this. If predation pressure from raccoons on rare native species increases during their spawning season, then this could directly affect their populations. Because the predation impacts of raccoons have not been accurately evaluated, it has been difficult to design effective strategies to control this invasive species. If the impacts of raccoon predation on rare native species are ignored, raccoons may negatively affect Japanese ecosystems. Therefore, we investigated the possible impacts of predation by analysing the feeding habits of raccoons and examining the presence of predation using DNA metabarcoding and extent of predation using stable isotope analyses.

## Results

### DNA metabarcoding

We conducted DNA metabarcoding analyses for samples S1–S5 and G1–G2 (Table 1). For the gSalamander primer, we obtained 92,885 MiSeq reads from the G-1 sample, of which 82,172 reads passed the quality control processes. Of these reads, 78,517 were assigned to known species with ≥ 97% identity to reference sequences in the database; these 78,517 reads were assigned to 9 operational taxonomic units (OTUs). For the gInsect primer, we obtained 166,813 MiSeq reads from the G-1, G-2 and S-2 samples, of which 102,966 reads passed the quality control processes. Of these reads, 84,638 were assigned to known species with ≥ 97% identity to reference sequences in the database; these 84,638 reads were assigned to 47 OTUs. For PCR targeting the cytochrome c oxidase subunit I (COI) region of mitochondria, we obtained 240,568 MiSeq reads from G-1, G-2, S-2 and S-3 samples, of which 135,943 reads passed the quality control processes. Of these reads, 54,682 were assigned to known species with ≥ 97% identity to reference sequences in the database; these 54,682 reads were assigned to 74 OTUs (Table 2). Hokkaido salamander and Hokkaido brown frog (*Rana pirica*) sequences were detected in the G-1 sample from raccoons captured in April (Table 3). Sequences of other animals were detected in some samples (Table 3), but Japanese crayfish sequences were not detected in any sample. No amplifications were successful for the rectal faeces samples S-1, S-4, and S-5 using the primers gSalamander and gInsect (Table 1).

**Table 1:** Raccoon samples used for DNA metabarcoding and results of DNA amplification.

Sample ID	No. of samples	Capture month	Primer name and results of amplification		
			gSalamander	gInsect	COI
S-1	4	May	×	×	×
S-2	3	May	−	○	○
S-3	2	April	−	×	○
S-4	5	July	×	×	Not conducted
S-5	4	August	×	×	Not conducted
G-1	2	April	○	○	○
G-2	4	May	×	○	○

○, specific animal species that could be a food resource was detected by sequence analysis; − , DNA was amplified, but animal species that could be a food resource was not detected; × , DNA was not amplified.

**Table 2:** Summary of Miseq reads.

Sample ID	Primer name	Raw data from Miseq	Passed reads of the quality control processes	With ≥ 97% identity to reference sequences	No. of OTUs
G-1	gSalamander	92,885	82,172	78,517	9

Sample ID	Primer name	Raw data from Miseq	Passed reads of the quality control processes	With ≥ 97% identity to reference sequences	No. of OTUs
G-1	glnsect	88,106	65,211	50,987	13
G-2	glnsect	17,643	6,912	6,807	3
S-2	glnsect	61,064	30,843	26,844	31
Total		166,813	102,966	84,638	47

Sample ID	Primer name	Raw data from Miseq	Passed reads of the quality control processes	With ≥ 97% identity to reference sequences	No. of OTUs
G-1	COI	57,190	32,752	9,258	14
G-2	COI	38,720	26,753	19,558	8
S-2	COI	80,731	39,456	16,108	37
S-3	COI	63,927	36,982	9,758	15
Total		240,568	135,943	54,682	74

**Table 3:** Animal species detected by DNA metabarcoding.

Species name	G-1							
	Number of sequence reads	Number of OTUs						
**Primer: gSalamander**								
*Hynobius retardatus*	61,428	4						
*Gadus chalcogrammus* (used as bait)	15,636	4						
*Procyon lotor*	1,453	1						
Total	78,517	9						

Species name	G-1		G2		S-2			
	Number of sequence reads	Number of OTUs	Number of sequence reads	Number of OTUs	Number of sequence reads	Number of OTUs		
**Primer: gInsect**								
*Yezoterpnosia nigricosta*	0	0	0	0	26,844	31		
*Curculio davidi*	35,735	7	0	0	0	0		
*Chironomidae*	15,252	6	0	0	0	0		
*Eubasilissa regina*	0	0	6,302	2	0	0		
*Procyon lotor*	0	0	505	1	0	0		
Total	50,987	13	6,807	3	26,844	31		

Species name	G-1		G2		S-2		S-3	
	Number of sequence reads	Number of OTUs	Number of sequence reads	Number of OTUs	Number of sequence reads	Number of OTUs	Number of sequence reads	Number of OTUs
**Primer: COI**								
*Hynobius retardatus*	1,337	1	0	0	0	0	0	0
*Rana pirica*	365	1	0	0	0	0	0	0
*Yezoterpnosia nigricosta*	0	0	0	0	14,955	34	0	0
*Armadillidium vulgare*	0	0	0	0	0	0	505	1
*Curculio sikkimensis*	491	1	0	0	0	0	0	0
*Eubasilissa regina*	0	0	16,320	3	0	0	149	1
*Lelia decempunctata*	109	1	0	0	0	0	0	0
*Limnophyes minimus*	415	1	0	0	0	0	0	0
*Megascolecidae* sp.	282	1	0	0	0	0	417	1
*Euhadra brandtii*	535	1	415	1	0	0	0	0
*Ezohelix gainesi*	0	0	388	1	0	0	0	0
Other species (Fungi etc.)	2,884	4	244	2	1,153	3	7,518	10
Other species (Fish and meat used as bait)	1,994	2	0	0	0	0	411	1
*Procyon lotor*	846	1	2,191	1	0	0	758	1
Total	9,258	14	19,558	8	16,108	37	9,758	15

### Stable isotope analysis

**Figure 2:**
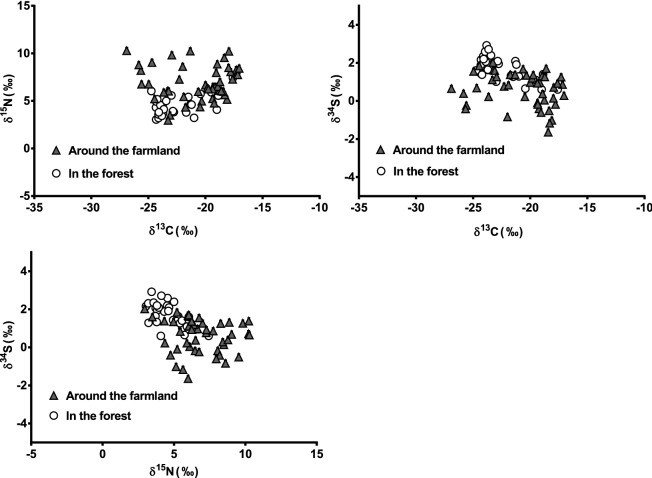
δ^13^C, δ^15^N, and δ^34^S values in thigh muscle samples of raccoons. Closed triangles indicate values for raccoons captured around the farmland; open circles indicate values for raccoons captured in the forest.

**Figure 3:**
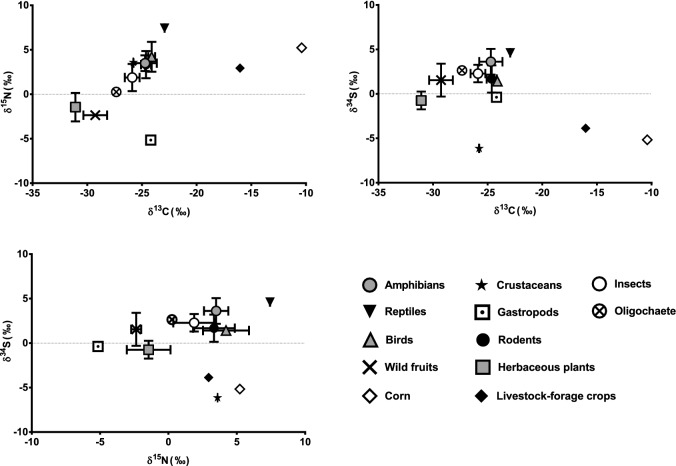
δ^13^C, δ^15^N, and δ^34^S values for potential prey items of raccoons. Symbols indicate mean ± SE.

We conducted stable isotope analyses of thigh muscle samples and potential prey items of raccoons (Fig. 2, Fig. 3). The δ^13^C, δ^15^N, and δ^34^S values for raccoons were − 21.44‰ ± 2.61‰ (average ± SD), 5.98‰ ± 1.91‰, and 1.04‰ ± 0.97‰, respectively. The analytical precision of δ^13^C, δ^15^N, and δ^34^S analyses were within ± 0.20‰, ± 0.20‰, and ± 0.30‰, respectively.

The results of the two-way ANOVA revealed significant main effects for capture site (around the farmland *vs.* in the forest) for δ^13^C, δ^15^N, and δ^34^S (δ^13^C: *F* (1,71) = 8.565, *P* < 0.05, δ^15^N: *F* (1,71) = 17.576, *P* < 0.05, δ^34^S: *F* (1,71) = 25.831, *P* < 0.05) (Table 4). No significant main effects were found for season (spring: April–June *vs.* summer: July–August) for δ^13^C, δ^15^N, and δ^34^S (δ^13^C: *F* (1,71) = 0.004, *P* = 0.952, δ^15^N: *F* (1,71) = 1.498, *P* = 0.225, δ^34^S: *F* (1,71) = 0.269, *P* = 0.606) (Table 4). No significant interaction effects were found between capture site and season for δ^13^C, δ^15^N, and δ^34^S (δ^13^C: *F* (1,71) = 0.909, *P* = 0.344, δ^15^N: *F* (1,71) = 0.105, *P* = 0.747*,* δ^34^S: *F* (1,71) = 1.212, *P* = 0.275) (Table 4). The results of the Mann–Whitney U test showed that, for capture sites, δ^13^C and δ^15^N were significantly higher in raccoons captured around the farmland than in those captured in the forest (δ^13^C: *P* < 0.01; δ^15^N: *P* < 0.001). However, δ^34^S was significantly higher in raccoons captured in the forest than in those captured around the farmland (δ^34^S: *P* < 0.001).

**Table 4:** Two-way ANOVA results.

	δ^13^C			δ^15^N			δ^34^S		
	DOF*	*F* value	*P* value	DOF	*F* value	*P* value	DOF	*F* value	*P* value
Capture site (around the farmland vs. in the forest)	1, 71	8.565	< 0.05	1, 71	17.576	< 0.05	1, 71	25.831	< 0.05
Season (spring vs. summer)	1, 71	0.004	0.952	1, 71	1.498	0.225	1, 71	0.269	0.606
Interaction effect (capture site × season)	1, 71	0.909	0.344	1, 71	0.105	0.747	1, 71	1.212	0.275

DOF* degree of freedom.

Among the potential food resources for raccoons, corn (− 10.39‰) and livestock forage crops (− 16.03‰) were very high in δ^13^C, whereas herbaceous plants (− 31.08‰ ± 0.42‰), wild fruits (− 29.27‰ ± 1.08‰), and oligochaete (earthworms) (− 27.35‰) were low in δ^13^C. The δ^15^N value was very high for reptiles (7.43‰), relatively high for corn (5.22‰) and birds (4.21‰ ± 1.68‰), but very low in gastropods (− 5.15‰), wild fruits (− 2.36‰ ± 0.37‰), and plants (− 1.45‰ ± 1.60‰). The δ^34^S values were high for reptiles (4.62‰) and amphibians (3.61‰ ± 1.44‰), relatively high for oligochaetes (2.63‰) and insects (2.28‰ ± 0.98‰), and low for crustaceans (Japanese crayfish) (− 6.14‰), corn (− 5.17‰), and livestock forage crops (− 3.87‰).

**Figure 4:**
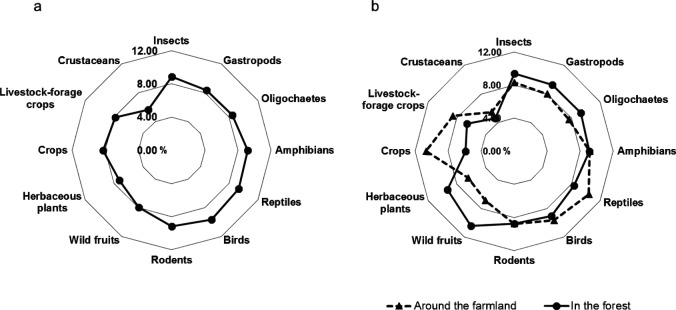
Foraging ratios of raccoons. (**a**) Foraging ratios of raccoons from late winter to early summer. Contribution of each food resource to raccoon diet was estimated using the Bayesian mixing model, IsoWeb. (**b**) Comparison of foraging ratios between raccoons captured around the farmland and those captured in the forest. Dotted line indicates ratios for raccoons captured around the farmland; solid line indicates ratios for raccoons captured in the forest.

We estimated the contribution of each food resource to the raccoon diet using IsoWeb (Fig. 4). In whole captured raccoons, the main animal food resources were birds (9.66%), reptiles (9.35%), rodents (9.18%), and amphibians (9.15%). Animal food resources accounted for 68.7% of total consumed food. We also compared the feeding habits between raccoons captured around the farmland and those captured in the forest. For the raccoons captured around the farmland, the main food sources were crops (10.65%), reptiles (10.45%), and livestock forage crops (8.56%). For those captured in the forest, the main food sources were wild fruits (10.41%), insects (9.42%), herbaceous plants (9.31%), and oligochaetes (9.30%). Raccoons from both types of capture sites showed similar foraging ratios of amphibians, including salamanders (about 9%), birds (about 10%), and rodents (about 9%). For animals from both types of capture sites, the lowest foraging ratio was crustaceans (Japanese crayfish) (around the farmland: 5.5%, in the forest: 4.6%).

## Discussion

In this study, we estimated the contribution of each food resource to the raccoon diet using δ^13^C, δ^15^N, and δ^34^S values measured for muscles of raccoons captured from April to August. As a result, animal food resources accounted for about 70% of the total consumed food, both for raccoons captured around the farmland and those captured in the forest. The main animal food resources were birds (9.66%), reptiles (9.35%), rodents (9.18%), and amphibians (9.15%). In our previous study on the diet of raccoons in Nopporo Forest Park from spring to summer, a faecal analysis showed that plant material food resources, such as herbaceous plants, crops and wild fruits, accounted for about 80% of total consumed food; vertebrates, such as amphibians, accounted for less than 2%^23^. The results of this study contradicted those of the conventional method, faecal analysis, because it is difficult to confirm the presence of easily digested animal food resources, such as amphibians and reptiles, in stomach contents and faeces^24^.

We compared the feeding habits between raccoons captured around the farmland and those captured in the forest. Of the plant food resources, crops and livestock forage crops were often consumed by raccoons around the farmland, whereas wild fruits and herbaceous plants were consumed more by raccoons in the forest (Fig. 4). Although raccoons show a strong preference for corn^25^, our results show that crop plants accounted for only 11% of the diet of raccoons captured around the farmland, which was not much different from the proportion of other food resources. In Hokkaido, corn generally matures after August, or July at the earliest for corn plants cultivated in greenhouses. Therefore, considering the turnover period of stable isotopes in muscle, the dietary information for corn will be reflected in stable isotope data for raccoons captured only from late July to August. When the abundance of food resources for raccoons increases in summer, they do not need to eat the food in the traps; therefore, they are less likely to be caught. In this study, only 4 out of 46 raccoons were captured around the farmland after mid-July, which explains the low foraging ratio of corn in raccoons captured around the farmland. The foraging ratio of animal food resources, such as amphibians, birds, rodents, and crustaceans, out of total consumed food was almost the same in both regions (around the farmland: 68%, in the forest: 67%). In both regions, the foraging ratio of amphibians, including salamanders, was about 9%, and that of crustaceans, including Japanese crayfish, was about 5%. The results suggested that raccoons prey on various animal food resources, including amphibians and crustaceans to the same extent, regardless of the habitat, from late winter to early summer. Our findings show that animal food resources are important for raccoons from late winter to early summer, when other food resources are depleted.

In this study, the δ^34^S values tended to be higher for forest resources than for crops and livestock forage crops and were very low in crustaceans (Japanese crayfish). Some crustaceans eat clams that are symbiotic with chemoautotrophic bacteria, which synthesise organic matter using reduced sulfur compounds^26^. Sulfate-reducing bacteria reduce sulfate ions (SO^4-^) to hydrogen sulfide (H_2_S), which lowers the value of the δ^34^S values^21^. Such sulfate-reducing bacteria are widely present in nature, such as in soils, rivers, streams, lakes, ponds, and paddy soils^27,28^. Although Japanese crayfish eat earthworms and insects, they rarely encounter these animal food resources. Their main food resources are humified fallen leaves and branches that cover their habitats^29^. However, because microorganisms are attached to fallen leaves, crayfish ingest the microorganisms as protein when they eat the fallen leaves^29^. It is possible that the δ^34^S value in Japanese crayfish was low because of its feeding situation.

Japanese crayfish were not detected by DNA metabarcoding. However, they were previously detected in the gastrointestinal contents of multiple raccoons in our study area^14^. They were also detected in the gastrointestinal contents of multiple raccoons captured in 2006 and 2008 (our unpublished data). A crayfish researcher successfully observed raccoons trying to prey on Japanese crayfish by camera trap survey in 2015 (Tanaka K., personal observation). Raccoons are known to prefer aquatic organisms such as amphibians and crayfish^5^. In our camera trap survey, raccoons were often observed locating food in streams using their forelimbs. From these data, it was determined that the raccoons inhabit Nopporo Forest Park are preying on Japanese crayfish. However, it is difficult to evaluate the size of the Japanese crayfish population.

A survey of Japanese crayfish distribution in 2003 showed that the Japanese crayfish population and the proportion of mature female individuals were smaller in Nopporo Forest Park than in western Hokkaido where raccoons had not yet invaded^30^. We also conducted a survey of Japanese crayfish by turning over stones, fallen trees, and fallen leaves in streams, and counting the number of Japanese crayfish. Accurate crayfish population surveys are very difficult because the number of crayfish found may fluctuate because of changes in the environment, such as water temperature on the day of the survey (Tanaka, K., personal observation). However, we inferred that the size of the Japanese crayfish population has decreased (our unpublished data). This observation has also been confirmed by a researcher who regularly surveys Japanese crayfish in the forest (Tanaka, K., personal observation).

We detected Hokkaido salamanders from the gastric contents of raccoons by DNA metabarcoding for the first time. In our study area, Nopporo Forest Park, Hori et al.^15^ successfully observed raccoons preying on Hokkaido salamanders by camera trap survey in spring. A previous camera trap survey revealed that the predator who left only the salamander's tail was a raccoon^15^. We have observed these tail remains of Hokkaido salamanders yearly in the spring (Sashika, M., personal observation). However, it was difficult to evaluate the size of the salamander population. One of the methods is counting the number of the salamander’s egg sacs. Because the female salamanders lay a pair of the egg sacs in still water, such as pools and small ponds, the number of females can be estimated by counting the egg sacs^31^. Hori et al.^15^ reported local reduction in the number of Hokkaido salamander egg sacs in Nopporo Forest Park. We also conducted a survey in which we counted the egg sacs and confirmed local reduction in the forest (our unpublished data).

We also detected Hokkaido brown frog, another Japanese native amphibian, in the same samples in which Hokkaido salamanders were detected. Hori et al.^15^ successfully observed raccoons preying on Hokkaido brown frog by camera trap survey in spring. Frog bones were detected in multiple individuals in our previous study^23^. Therefore, frog bones can sometimes be detected in the faeces or stomach contents of raccoons. However, when we directly observed the samples, we did not detect any frog bones. Our results suggest that raccoons prey on animal food resources such as amphibians more than has been estimated by conventional analyses of stomach contents and faeces. It is difficult to determine how much of the total amount of amphibians that Hokkaido salamanders account for in the raccoon's diet because Hokkaido salamanders and Hokkaido brown frogs have similar δ^13^C, δ^15^N, and δ^34^S values. However, it can be inferred that Hokkaido salamanders and Hokkaido brown frogs are important food resources for raccoons by studies conducted to date. The spawning season of Hokkaido salamanders and Hokkaido brown frog is from April to May, and many individuals gather at one breeding site to spawn^16,31^. Both amphibians usually use the same spawning sites in still water, such as pools and small ponds ^31,32^. Raccoons have a very high learning ability, and once they find efficient food resources, they repeatedly forage similar food resources^33^. Therefore, during seasons when raccoons’ other food resources are depleted, both amphibians are very easily predated on by raccoons. Although Hokkaido brown frog is not currently specified as a noteworthy species, the impact of raccoons should be carefully investigated in the future.

Our study reveals that nutritious amphibians and Japanese crayfish are important food resources for raccoons from late winter to early summer when other food resources are depleted. In addition, such selective predation pressure on Japanese crayfish and Hokkaido salamander during their spawning season by raccoons can directly affect isolated populations such as those in Nopporo Forest Park^33^. If the population density of raccoons increases, the predation pressure on rare native species during their spawning season may also increase.

It remains unclear whether the abundance of rare native species is mainly affected by raccoons, or other factors such as environmental changes. Therefore, further studies are necessary to investigate the impacts of other factors and changes on the populations of rare native species. It is also important to determine the status (presence or absence) of Japanese crayfish and Hokkaido salamanders in habitats using environmental DNA analysis in the future.

By conducting DNA metabarcoding analyses, we detected DNA of readily digested animal materials, which are difficult to detect by conventional methods. However, we could not amplify the DNA from two rectal faeces samples collected in July and August by PCR because the temperatures were higher than in April and May and the samples had deteriorated. In further studies, it will be useful to analyse more samples to gain a more detailed understanding of the raccoon diet. In addition, because we added measurements of δ^34^S, the accuracy of discrimination between food resources was improved, and we could estimate the feeding habits of raccoons more precisely. The conventional method of measuring only δ^13^C and δ^15^N can distinguish between forest resources and agricultural-based food resources such as corn and livestock forage crops^23^. However, food resources with similar δ^13^C and δ^15^N values, such as amphibians, reptiles, birds, rodents, and crustaceans, are more difficult to distinguish. Therefore, conventional methods alone are insufficient to accurately estimate the impacts of alien species. In the future, as in our research, it will be important to add and combine new methods to determine the impacts of alien species on native species. Our study significantly contributes to the conservation of rare aquatic small organisms and promotes raccoon control measures.

## Conclusion

Our study reveals that nutritious animal food resources, including amphibians and crustaceans, are important for raccoons from late winter to early summer, when other food resources are depleted. Using a DNA-based approach, Hokkaido salamanders were detected in the gastrointestinal contents of raccoons. In addition, by conducting a sulfur stable isotope analysis, we were able to more precisely estimate raccoon feeding habits than would be possible using a conventional stable isotope analysis based only on carbon and nitrogen. As a result, raccoons have been found to use amphibians at a higher rate than previously reported, including a rare endangered species, the Hokkaido salamander. The results also show that there is some predation on Japanese crayfish by raccoons. Both Hokkaido salamander and Japanese crayfish spawn in spring. If predation pressure on rare native species by raccoons is increased during their spawning season, then raccoons will directly impact the populations of these species.

## Methods

### Study area

We conducted our study at Nopporo Forest Park in Hokkaido, Japan (43° 03′ N, 141° 32′ E) (Fig. 1). This forest is a semi-isolated 2,053 ha area surrounded by residential areas and farmland. There have been concerns about raccoon impacts on this ecosystem since they were first detected in 1992^34^.

### Animals

We captured raccoons using box traps (Havahart Large Collapsible Pro Cage Model 1089, Woodstream Corp., Lititz, PA, USA) from May to July in 2018 and April to August in 2019. Traps were placed at 86 sites (Fig. 1). Trapping points within 250 m of the forest boundary line facing the farmland were defined as ‘around the farmland’ (31 points), and other points were defined as ‘in the forest’ (55 points) using QGIS version 3.10^23^. Captured raccoons were anesthetised with butorphanol tartrate (Vetorphale 5 mg, 1.2 mg/kg; Meiji Seika, Tokyo, Japan), medetomidine hydrochloride (Dolbene, 40 µg/kg; Kyoritsu, Tokyo, Japan), and midazolam (Dormicum injection 10 mg, 0.2 mg/kg; Astellas, Tokyo, Japan) by intramuscular injection and euthanised by potassium chloride injection into the heart. We captured 48 raccoons (34 around the farmland, 14 in the forest) in 2018, and 27 (12 around the farmland, 15 in the forest) in 2019 (Fig. 1). We collected thigh muscle samples, rectal faeces, and gastric contents from captured raccoons. Samples were stored at − 20 °C until analysis.

### DNA metabarcoding

#### Sample preparation

Rectal faeces and gastric contents that were dominated by plant materials and groomed hair were excluded from analyses. In total, 18 rectal faeces and six gastric content samples that contained animal materials or whose contents were unknown were selected. Rapidly digested food resources such as amphibians cannot be confirmed visually in gastrointestinal contents, and even DNA may not be detected. Therefore, multiple samples were pooled and analysed because the target DNA may not be detected otherwise. The rectal faeces and gastric contents were divided into five (S1–S5) and two groups (G1–G2), respectively, based on the capture site and date of raccoons (Table 1). Each group was mixed and stored at − 20 °C until DNA extraction. Subsequent DNA analyses were performed at Bioengineering Lab. Co., Ltd. (Kanagawa, Japan).

#### DNA extraction

Samples (about 100 mg each) were lyophilised using a VD-250R lyophiliser freeze dryer (TAITEC, Saitama, Japan) and ground using a ShakeMaster NEO homogeniser (Bio Medical Science, Tokyo, Japan). Crude DNA was extracted from each group; then, DNA was purified using the MPure-12 Automated Nucleic Acid Purification System (MP Biomedicals, California, USA) with a MPure Bacterial DNA Extraction Kit (MP Biomedicals).

#### Library preparation and sequencing

Each library was prepared using two-step tailed PCR. We used the gSalamander primer, which amplifies salamander and newt 12S rRNA, as a specific primer to detect Hokkaido salamander, and the gInsect primer, which amplifies arthropod 16S rRNA, to detect Japanese crayfish (Table 5). All primers were designed by Bioengineering Lab. Co., Ltd.

**Table 5:** Primer sets used in this study.

Name of primer	Target Taxa	Gene	Primer sequence (5′ → 3′)
1st-gSalamanderF	Salamanders and Newts	12S rRNA	ACACTCTTTCCCTACACGACGCTCTTCCGATCTNNNNNNCACCGCGGTTATACGAGARAC
1st-gSalamanderR			GTGACTGGAGTTCAGACGTGTGCTCTTCCGATCTNNNNNNGCACCGCCAAGTCCTTTGAG
1st-gInsectF	Arthropods	16S rRNA	ACACTCTTTCCCTACACGACGCTCTTCCGATCTNNNNNNGATAGAAACCAACCTGGCT
1st-gInsectR			GTGACTGGAGTTCAGACGTGTGCTCTTCCGATCTNNNNNNGACGAGAAGACCCTATA
1st-IntF	Animals	Cytochrome c Oxidase subunit I (COI)	ACACTCTTTCCCTACACGACGCTCTTCCGATCTGGWACWGGWTGAACWGTWTAYCCYCC
1st-HCOmR			GTGACTGGAGTTCAGACGTGTGCTCTTCCGATCTTAHACTTCNGGGTGKCCRAARAATCA
2ndF			AATGATACGGCGACCACCGAGATCTACAC-Index1-ACACTCTTTCCCTACACGACGC
2ndR			CAAGCAGAAGACGGCATACGAGAT-Index2-GTGACTGGAGTTCAGACGTGTG
Procyon lotor-COI-Blocking-primer			CCTCCTCTAGCAGGTAACCTAGCACATGC /3SpC3/

Index1 and Index2 indicate sample identification number.

The first PCR amplified the target region using gSalamander and gInsect. These reactions were conducted in a final volume of 10 μl, comprising 2 μl DNA template, 0.5 μl each primer (10 μM), 0.1 μl Ex Taq HS (5 U/µl) (Takara Bio Inc., Shiga, Japan), 1.0 μl 10 × Ex Taq buffer, 0.8 μl dNTP mixture (2.5 mM), and 5.1 μl sterile distilled water. The PCR conditions were: first denaturation for 2 min at 94 °C, followed by 35 cycles of 30 s at 94 °C, 30 s at 50 °C, and 30 s at 72 °C, and final extension for 5 min at 72 °C.

The second PCR used the first PCR products as the template with index primers (2ndF and 2ndR). These reactions were conducted in a final volume of 10 μl, as described for the first PCR. The PCR conditions were: first denaturation for 2 min at 94 °C, followed by 12 cycles of 30 s at 94 °C, 30 s at 60 °C, and 30 s at 72 °C, and final extension for 5 min at 72 °C. At each step, PCR products were purified using the Agencourt AMPure XP system (Beckman Coulter, Inc., California, USA). The library concentrations were measured with a Synergy H1 microplate reader (BioTek) and a QuantiFluor dsDNA System (Promega). Library quality was assessed using a Fragment Analyser (Advanced Analytical Technologies, Iowa, USA) with a dsDNA 915 Reagent Kit (Agilent, California, USA). Paired-end sequencing (2 × 300 bp) was conducted on the Illumina MiSeq platform (Illumina, California, USA).

#### Data analysis

Reads that began with a sequence that completely matched the primer used were extracted using the fastq_barcode_splitter tool in the FASTX-Toolkit; then, the primer sequence was trimmed. The reads were trimmed and filtered using the Sickle tool with a quality value of 20; then, trimmed and paired-end reads with fewer than 150 bases were discarded. The remaining reads were merged using the FLASH paired-end merge script^35^ under the following conditions: fragment length after merge, 300 bases; read fragment length, 230 bases; and minimum overlap length, 10 bases. The UCHIME2 algorithm within USEARCH was used to check all filtered sequences for chimeric sequences^36^. All sequences that were not judged to be chimeras were used for further analysis. The UPARSE algorithm within USEARCH was used for OTU creation and taxonomic assignments. The constructed OTUs were subjected to Basic Local Alignment Search Tool (BLASTN) searches. More than 100 reads and the top BLAST hit with a sequence identity of ≥ 97% were used to assign species (target length: about 300 bp) to each representative sequence^37^.

#### Additional analyses of COI region

S–2, S–3, G–1, and G–2 sample group DNA was successfully extracted using gSalamander or gInsect primers (Table 1) and analysed by PCR using COI and blocking primers for raccoon (Table 5). The library was prepared using two-step tailed PCR. The first PCR amplification using the primer set 1st-IntF and 1st-HCOmR was conducted in a final volume of 10 μl, comprising 2 μl DNA template, 5 μl of each primer (10 μM) (forward primer 0.5 µl, reverse primer 0.5 µl, blocking primer 4 µl), 0.08 μl Ex Taq HS (5 U/µl), 1.0 μl 10 × Ex Taq buffer, 0.8 μl dNTP mixture (2.5 mM), and 1.12 μl DDW. The PCR conditions were as follows: first denaturation for 2 min at 94 °C, followed by 35 cycles of 30 s at 94 °C, 15 s at 67 °C, and 30 s at 52 °C and 30 s at 72 °C, and final extension for 5 min at 72 °C. Subsequent methods were as described above, except for the FLASH paired-end merge script (fragment length after merge, 310 bases; read fragment length, 225 bases).

#### Stable isotope analysis

Stable isotope ratios of muscle tissue reflect the diet over the previous few weeks to one month^38,39^. We used the muscles of raccoons captured from April to August and assumed that the stable isotope ratios in raccoon muscle samples reflected their diet from March to July, i.e. late winter to early summer, in Hokkaido.

Raccoon muscle and potential prey item samples were dried at 60 °C for > 24 h and then ground with a mortar and pestle. Potential food items (such as amphibians and crustaceans) were collected from the forest. The raccoon muscle and potential prey item samples were rinsed with a 2:1 chloroform: methanol solution to remove lipids and then dried at 60 °C for at least 24 h^40^. Each sample (1.0–3.0 mg) was enclosed in a tin cup and combusted in an elemental analyser (Vario MICRO cube, Elementar Gmbh, Hanau, Germany) interfaced with an isotope ratio mass spectrometer (IsoPrime100, Elementar Gmbh). We determined the δ^13^C, δ^15^N, and δ^34^S values for each sample. The results are reported as parts per thousand of the isotopes relative to a standard. For δ^13^C, δ^15^N, and δ^34^S values, Vienna Pee Dee Belemnite, air, and Vienna Cañon Diablo Triolite were used as standards, respectively. We used L-alanine (Shoko Science Co., Ltd., Tokyo, Japan) and sulfanilamide (Elementar GmbH) as working standards. A working standard, sulfanilamide (δ^34^S value, − 1.92‰), was calibrated against IAEA (International Atomic Energy Agency, Vienna, Austria) silver sulfides, IAEA-S-1, IAEA-S-2, IAEA-S-3, and was used as a working standard for δ^34^S.

#### Statistical analysis

We performed two-way analysis of variance to examine the interactions between two independent variables, season (spring: April–June vs. summer: July–August) and capture site (around the farmland vs. in the forest), and their relationship with the dependent variables (stable isotope ratio; δ^13^C, δ^15^N, and δ^34^S). After examining interactions between two independent variables (season and capture site), a Mann–Whitney U test was conducted. Differences were considered statistically significant at *P* < 0.05.

We estimated the contribution of each food resource to the raccoon diet using IsoWeb^22^. This analysis was performed with the trophic enrichment factors of the δ^13^C, δ^15^N, and δ^34^S values set to 1.1‰, 3.4‰, and 0.5‰, respectively^41,42^.

SPSS Statistics 20.0 (IBM, Tokyo, Japan) and R (R Core Development Team, R Foundation for Statistical Computing, Vienna, Austria) were used for all statistical analyses.

### Ethical approval

All procedures were conducted in accordance with the Guidelines for Animal Care and Use of Hokkaido University and were approved by the Animal Care and Use Committee of the Faculty of Veterinary Medicine, Hokkaido University (Permit Number: 18-0001). Permission to capture raccoons was obtained from the Ministry of the Environment, as part of the feral raccoon control program.

## Data Availability

All data needed to evaluate the conclusions in the paper are present in the paper.

## References

[1] Ikeda T, Yamada F, Ikeda T, Ogura G (2011). Invasive alien mammals in Japan. Invasive Alien Mammals in Japan.

[2] Secretariat of the Convention on Biological Diversity. Global Biodiversity Outlook 2. https://www.cbd.int/doc/gbo/gbo2/cbd-gbo2-en.pdf (2006).

[3] Gehrt S, Feldhamer G, Thompson B, Chapman J (2003). Raccoons and allies. Wild Mammals of North America.

[4] Harman, D. M. & Stains, H. J. The raccoon (*Procyon lotor*) on St. Catherines Island, Georgia. 5, Winter, spring, and summer food habits. *Am*. *Mus*. *Novit*. **2679** (1979).

[5] Goldman EA, Jackson HHT (1950). Raccoons of north and middle America. North Am. Fauna..

[6] Stuewer FW (1943). Raccoons: their habits and management in Michigan. Ecol. Monogr..

[7] Llewellyn LM, Uher FM (1952). The foods of fur animals of the Patuxent Research Refuge, Maryland. Am. Midl. Nat..

[8] Johnson AS (1970). Biology of the raccoon (*Procyon lotor varius*) in Alabama. Ala. Agr. Exp. Sta. Repo..

[9] Ikeda T, Asano M, Matoba Y, Abe G (2004). Present status of invasive alien raccoon and its impact in Japan. Glob. Environ. Res..

[10] Ikeda T, Takatsuki S, Yamagiwa J (2008). Invasive alien species issues, with special reference to raccoons. Mammalogy in Japan.

[11] Abe G, Yamada F, Ikeda T, Ogura G (2011). Raccoon. Invasive Alien Mammals in Japan.

[12] Tokuda, T. Hokkaido salamander. In *Compact picture guide to Hokkaido reptiles and amphibians*. 60–65 (Hokkaido News Press, 2011).

[13] Kawai, T. & Takahata, M. Physiology and ecology of Japanese crayfish. In Biology of crayfish. 362–381 (Hokkaido University Press, 2010).

[14] Hori S, Matoba Y (2001). Arthropods recognised from the contents in the digestive tract of raccoons. B. Hist. Mus. Hokkaido..

[15] Hori S, Ueki R (2013). Predation of native amphibians by raccoon (*Procyon lotor*) has been confirmed in Nopporo Forest Park. Hokkaido Herpetol. Soci..

[16] Sato, T. & Matsui, M. Ecology of Salamanders in *Salamanders of Hokkaido.* 34–69 (Eco-network, 2013).

[17] Minagawa M, Wada E (1984). Stepwise enrichment of δ^15^N along food chains: further evidence and the relation between δ^15^N and animal age. Geochim. Cosmochim. Ac..

[18] DeNiro MJ, Epstein S (1978). Influence of diet on the distribution of carbon isotopes in animals. Geochim. Cosmochim. Ac..

[19] Rau GH (1983). Animal ^13^C/^12^C correlates with trophic level in pelagic food webs. Ecology.

[20] Fry B, Sherr EB (1984). δ^13^C measurements as indicators of carbon flow in marine fresh water ecosystems. Contrib. Mar. Sci..

[21] Richards MP, Fuller BT, Sponheimer M, Robinson T, Ayliffe L (2003). Sulphur isotopes in palaeodietary studies: a review and results from a controlled feeding experiment. Int. J. Osteoarchaeol..

[22] Kadoya T, Osada Y, Takimoto G (2012). IsoWeb: a bayesian isotope mixing model for diet analysis of the whole food web. PLoS ONE.

[23] Osaki A (2019). Comparison of feeding habits and habitat use between invasive raccoons and native raccoon dogs in Hokkaido, Japan. BMC Ecol..

[24] Fukue Y, Takeshita T, Nakanishi N (2011). Diet analysis methods to assess the food habits of carnivore in Japan–I. Canidae, Mustelidae. Felidae. Mammal. Sci..

[25] Dunn JP, Chapman JA (1983). Reproduction, physiological responses, age structure, and food habits of raccoon in Maryland, USA. Z. Säugetierkunde..

[26] Higgs ND, Newton J, Attrill MJ (2016). Caribbean spiny lobster fishery is underpinned by trophic subsidies from chemosynthetic primary production. Curr. Biol..

[27] Matsui S, Tatewaki M (1989). Sulfate-reducing bacteria. Environ. Tech..

[28] Furusaka C (1968). Studies on the activity of Sulfate-reducing bacteria in paddy soil. B. Inst. Agri. Res. Tohoku Univ..

[29] Kawai T, Hamano T, Matsuura S (1995). Feeding behaviour of the japanese crayfish *Cambaroides japonicas* (Decapoda, Astacoidea) in a stream in Hokkaido, Japan. Fish. Sci..

[30] Kawai T, Hori S, Mizushima M, Nagayasu Y (2004). Current status of distribution and population of Japanese crayfish in Nopporo Forest Park. B. Hist. Mus. Hokkaido..

[31] Kadosaki M (1997). The breeding of *Hynobius retardatus* and *Rana pirica* on Nopporo Forest park. J. Jpn. Wildl. Res. Soc..

[32] Sato T (1990). Temperature and velocity of water at breeding sites of *Hynobius retardatus*. J. J. Herpetol..

[33] Hori, S. & Mizushima, M. Amphibians in Nopporo Forest Park, Hokkaido, Japan. *B. Hist. Mus. Hokkaido.* 21–26 (2002).

[34] Kadosaki, M. Morphology and ecology of Hokkaido mammals in *Wildlife traces, 1st edn*. 370–372 (Hokkaido Publishing & Planning Center, 1996).

[35] Magoc T, Salzberg SL (2011). FLASH: fast length adjustment of short reads to improve genome assemblies. Bioinformatics.

[36] Edgar RC (2016). UCHIME2: improved chimera prediction for amplicon sequencing. bioRxiv.

[37] Kamimura S (2018). Fish diversity detection at port and urban canal area using environmental DNA mettabarcoding. J. Jap. Soc. Civ. Eng. (Ocean Eng.).

[38] Tieszen L, Boutton T, Tesdahl K, Slade N (1983). Fractionation and turnover of stable carbon isotopes in animal tissues: implications for δ^13^C analysis of diet. Oecologia.

[39] Sponheimer M (2006). Turnover of stable carbon isotopes in the muscle, liver, and breath CO_2_ of alpacas (*Lama pacos*). Rapid. Commun. Mass. Sp..

[40] Mizukami RN, Goto M, Izumiyama S, Hayashi H, Yoh M (2005). Estimation of feeding history by measuring carbon and nitrogen stable isotope ratios in hair of Asiatic black bears. Ursus.

[41] Roth JD, Hobson KA (2000). Stable carbon and nitrogen isotopic fractionation between diet and tissue of captive red fox: implications for dietary reconstruction. Can. J. Zool..

[42] McCutchan JH, Lewis WM, Kendall C, McGrath CC (2003). Variation in trophic shift for stable isotope ratios of carbon, nitrogen, and sulfur. Oikos.

